# Effects of Cardiorespiratory Exercise on Cognition in Older Women Exposed to Air Pollution

**DOI:** 10.3390/ijerph16020245

**Published:** 2019-01-16

**Authors:** Edgardo Molina-Sotomayor, Marcelo González Orb, Francisco Pradas de la Fuente, Giovanni Carozzi Figueroa, Antonio Jesús Sánchez-Oliver, José Antonio González-Jurado

**Affiliations:** 1 Departamento de Educación Física, Universidad Metropolitana de Ciencias de la Educación, Santiago 7760197, Chile; edgardo.molina@umce.cl (E.M.-S.); smgorb@yahoo.es (M.G.O.); 2 Facultad de Ciencias Humanas y de la Educación, Universidad de Zaragoza, Huesca 22003, Spain; franprad@unizar.es; 3 Departamento de Educación Física, Universidad San Sebastián-Chile, Recoleta, Santiago 8420000, Chile; alerceaustral@gmail.com; 4 Facultad de Ciencias del Deporte, Universidad Pablo de Olavide, Sevilla 41013, Spain; asanchez@upo.es; 5 Área de Motricidad Humana y Rendimiento Deportivo, Universidad de Sevilla, Sevilla 41013, Spain; asanchez38@us.es

**Keywords:** physical activity, cardiovascular, mental health, fitness, environmental health

## Abstract

The aim was to analyze the effects of cardiorespiratory exercise and air pollution on cognition and cardiovascular markers in four groups of older women: the active/clean air group (AC), the active/polluted air group (AP), the sedentary/clean air group (SC), and the sedentary/polluted air group (SP). Active groups performed a training task based on progressive walking. Prior to and after the experiment, the following parameters were assessed: cognition, by Mini Mental State Examination (MMSE); maximum oxygen uptake (VO_2max_), estimated by the Six-Minute Walk Test (6mWT); heart rate (HR); and oxygen saturation (SpO_2_). There were significant differences (*p* < 0.05) between the AC and the SP in all the MMSE dimensions except “Registration”, and in all the physiological variables (VO_2max_, SpO_2_, HR). Aerobic exercise may be a protective factor against the effects that pollution have on cognition and on the mechanisms of oxygen transport.

## 1. Introduction

Mental health and social relationships are determining factors of the quality of life of older people [1]. Air pollution not only increases the risk of cephalalgia (headache) in its population [2], but it also seems to be a risk factor of venous thrombosis and pulmonary embolism and has a high mortality rate [3]. It has been reported that air pollution by heavy metals has deleterious effects on the brain and central nervous system. [4]. It is known that long-term adverse effects are related to the processes of inflammation, coagulation [3], and oxidative stress that affect the functioning of the central nervous and cognitive systems [5]. Likewise, older people who live in areas of high atmospheric pollution have shown a generally lower performance in cognitive tests of verbal learning and memory, psychomotor speed, language, and executive function [6], demonstrating that exposure to air pollution may lead, in the long term, to significant cognitive impairment in older women [7]. The existing proof generally supports the association of adverse environmental factors with the mental health of the population [8].

On the other hand, it has been demonstrated that regular physical exercise improves cognition and reported evidence suggests that its effects on brain-derived neurotrophic factors (BDNFs) play an important role [9]. In older people with dementia subjected to cognitive activity combined with physical exercise, positive results were obtained for balance, memory, and quality of life relevant to daily living [10]. It has been reported that moderate cardiorespiratory aerobic activity is sufficient for the improvement of the cognitive function [11], especially in executive control, as it increases brain activity in the prefrontal and temporal regions and the volume of gray matter [12]. However, in young adults the effects of a program of cardiovascular aerobic exercise produced improvements in visuospatial memory but not in verbal memory [13]. On the other hand, this form of aerobic training performed within an urban environment with high air pollution has been related to an increase of inflammatory biomarkers, with no improvements in cognitive performance [14].

Exposure to particles such as ozone or carbon monoxide during exercise has harmful and systemic effects on pulmonary, cardiovascular, and cognitive functions (affecting oxygen consumption and sports performance [15]); there is evidence that demonstrates a greater relationship between brain activity during cognitive tasks and the level of physical cardiorespiratory fitness in healthy older people, with effects being attenuated in people with Alzheimer´s disease [16].

It has been mentioned that those who have lived in areas exposed to volcanic eruptions show lower peripheral oxygen saturation (SpO_2_) as a result of the effects of a high exposure to ultrafine pollutant particles (particulate material diameter ≤2.5 µm, PM_2.5_) [17]. The effects of aerobic cardiorespiratory training on the mental health of people who live in areas with high environmental pollution are still unknown. In this sense, the aim of the present study was to determine the chronic effects of cardiorespiratory physical activity on cognition and cardiovascular biomarkers in older women who live in areas of high air pollution.

## 2. Materials and Methods

**Figure 1:**
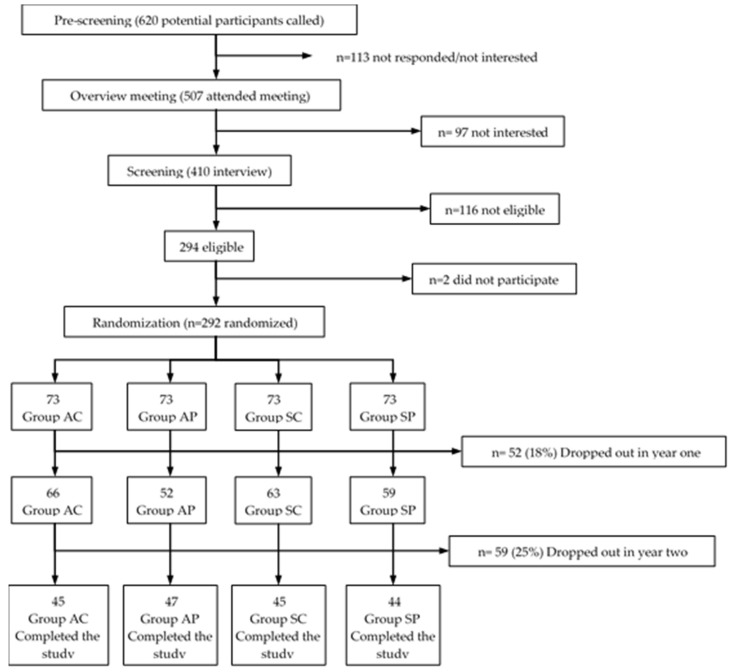
Sequence for recruitment screening. AC: active/clean air group; AP: active/polluted air group; SC: sedentary/clean air group; SP: sedentary/polluted air group.

The present work was a quasi-experimental, non-probabilistic prospective study that lasted two years. The participants (Figure 1) were recruited from a potential population of older women (*n* = 620), patients of the “La Estrella” Health Center of the Pudahuel commune, Metropolitan Region of Santiago de Chile (groups exposed to high air pollution), and patients of the Senior Centers of Viña del Mar City-Chile (groups exposed to low air pollution). A random initial sample (*n* = 292) divided into four groups was obtained. The final sample (*n* = 181) at the end of the experiment was constituted by the following groups: the active/polluted air group (AP, *n* = 47), age (X ± SD) 69.8 ± 4.3 years; the sedentary/polluted air group (SP, *n* = 44), age 68.3 ± 3.5 years; the active/clean air group (AC, *n* = 45) age 69 ± 1.2 years; and the sedentary/clean air group (SC, *n* = 45) age 68.5 ± 2.4 years.

The sample size was calculated with a 5% α and a 95% confidence level. The women of all four groups showed a level education that allowed them to read and write without any help. The experiment period began in March 2013 and ended in December 2014. 

Cognitive impairment was assessed through the Mini Mental State Examination (MMSE) [18]. The Spanish version was validated in a heterogeneous sample of older Chilean people, with a cutoff score for the diagnosis of mild cognitive impairment (MCI) ≤ 21. For higher educational levels, the use of a cutoff score between 23 and 24 has been suggested [19]. In the present study, due to the homogeneity of the educational level of the sample, a total valuation of 30 points was used, with a cutoff score for the diagnosis of pathological suspicion or MCI ≤ 24 [20].

The Six-Minute Walk Test (6mWT) allowed for the determination of cardiorespiratory performance (maximum oxygen uptake, VO_2max_) [21], since it provides reliable and valid measurements to assess the physical fitness related to health by predicting VO_2max_, expressed in liters O_2_/min, from a sub-maximal effort in a population without disabilities [22].

Two readings of heart rate (HR) and two of peripheral oxygen saturation (SpO_2_) were obtained prior to the experimental experiment (pretest): evaluation at rest (HRpre-1 and SpO_2_pre-1) and immediately after the Six-Minute Walk Test (HRpre-2 and SpO_2_pre-2). These two measurements were repeated two years after of the implementation of this program of aerobic physical exercise (post-test): at rest (HRpost-1 and SpO_2_post-1), and after sub-maximal effort through 6mWT (HRpost-2 and SpO_2_post-2).

HR and SpO_2_ levels were recorded using a pulse oximeter (Onyx 9500, onin®, Nonin Medical Inc, Plymouth, Minesota, US), a technique validated for first-line early detection and beneficial for indirect measurements of SpO_2_ at rest [23]. The measurements of the index with participant sitting were aseptically performed at an average environmental temperature in Viña del Mar city of 15 °C, with an atmospheric pressure of 1011.6 hPa at 17 m above sea level (a.s.l.), and in Pudahuel/Santiago at an average temperature of 16.7 °C, with an atmospheric pressure of 1013.2 hPa at 550 m a.s.l.

The inclusion criteria were: (1) women between 60 and 75 years of age, with up-to-date biochemical tests; (2) medical clearance; (3) older women of the Health Center of the Pudahuel commune-Metropolitan Region and Senior Centers of Viña del Mar city; (4) patients who had not performed any planned physical activity in the last 5 years; (5) patients who had lived not less than 30 years in their current homes; (6) patients with complete basic education who could read and write; (7) a score in the MMSE ≥ 25 points; (8) patients who used non-pollutant heating systems in their homes; and, (9) informed consent signed by the patient.

The exclusion criteria were: (1) patients with depression under treatment with antidepressants; (2) patients who showed pathological conditions that were incompatible with physical exercise; (3) cardiovascular diseases; (4) illiteracy; (5) smokers; (6) patients who left their current home for more than 3 months per year; (7) patients who attended less than 80% of the sessions of the training program; (8) patients who showed serious pathologies during the study period; and (9) incomplete measurements.

The experiment design followed the guidelines set by the Declaration of Helsinki, approved by the World Medical Association [24] and was approved by the Ethics Committee of Pablo de Olavide University (Seville, Spain).

Table 1 shows the characteristics of the cardiorespiratory training program. The exercise protocol consisted of low/moderate intensity walking on a horizontal plane, with progressive speed increases. The study duration was 24 months, with four training cycles of six months each and three 1-hour sessions per week. The training intensity was adjusted with VO_2max_ estimated by the 6mWT pretest.

Within the two-year period of the experiment, 11.7% of the sessions of physical exercise training planned to be performed outdoors were substituted with other equivalent indoor physical activities, following preventive environmental alerts due to the high average concentrations of particulate matter (PM_10_), which were above 200 µg/m^3^ in 24 h.

For the statistical analysis, the IBM SPSS 21 software was used (SPSS Inc., Chicago, IL, USA). The mean and standard deviation descriptive statistics were calculated. A confidence interval of the mean values was obtained at 95% to estimate the reliability of the measurements. With regard to inferential statistics, a multivariate general lineal model was applied, with the group factor, to analyze the differences between groups for both physiological and cognitive variables. The differences in comparisons by pairs (pairwise comparisons) were analyzed by the Bonferroni correction. Likewise, in order to determine the magnitude of the differences between the groups, the effect size was measured through partial eta squared. For the intragroup comparisons, the Student’s *t*-test or Wilcoxon test was conducted for related samples depending on the Shapiro–Wilk normality test.

## 3. Results

Table 2 shows the average triennial air pollution of the sample´s origin sites and the reference limit values of Chile and the World Health Organization (WHO).

Table 3 shows the comparisons, between groups, of the results obtained for the physiological variables and evaluation of the cognitive state through the MMSE at the beginning of the experiment (pre-test). The mean values of the four groups at the beginning of the experiment indicate no statistically significant differences between the groups (*p* > 0.05) and a small effect size in all the variables analyzed. Therefore, post hoc pairwise comparisons do not present significant differences. This clearly shows that there are no statistically significant differences among the different groups at the pre-tests.

Table 4 shows the comparisons, between groups, of the results obtained at the end of the experiment. Significant differences were found between groups in all the physiological variables analyzed. Post hoc analysis carried out shows pairwise comparisons. The same superscripts (^a^, ^b^, ^c^, ^d^, ^e^, ^f^) in values indicate significant differences between the groups. The active group who lived in a clean air environment (AC) obtained the best results in all the variables, and in the pairwise comparisons the differences with the two inactive groups (SC and SP) were statistically significant. In addition, Table 4 shows the comparisons, between the groups of the results obtained from the evaluation of the cognitive state of the participants through the MMSE and the corresponding dimensions at the end of the two-year period of the experiment. The two inactive groups (SC and SP) recorded lower values than the two active groups (AC and AP), both in the global valuation and in each of the indicative dimensions of the cognitive state.

**Figure 2:**
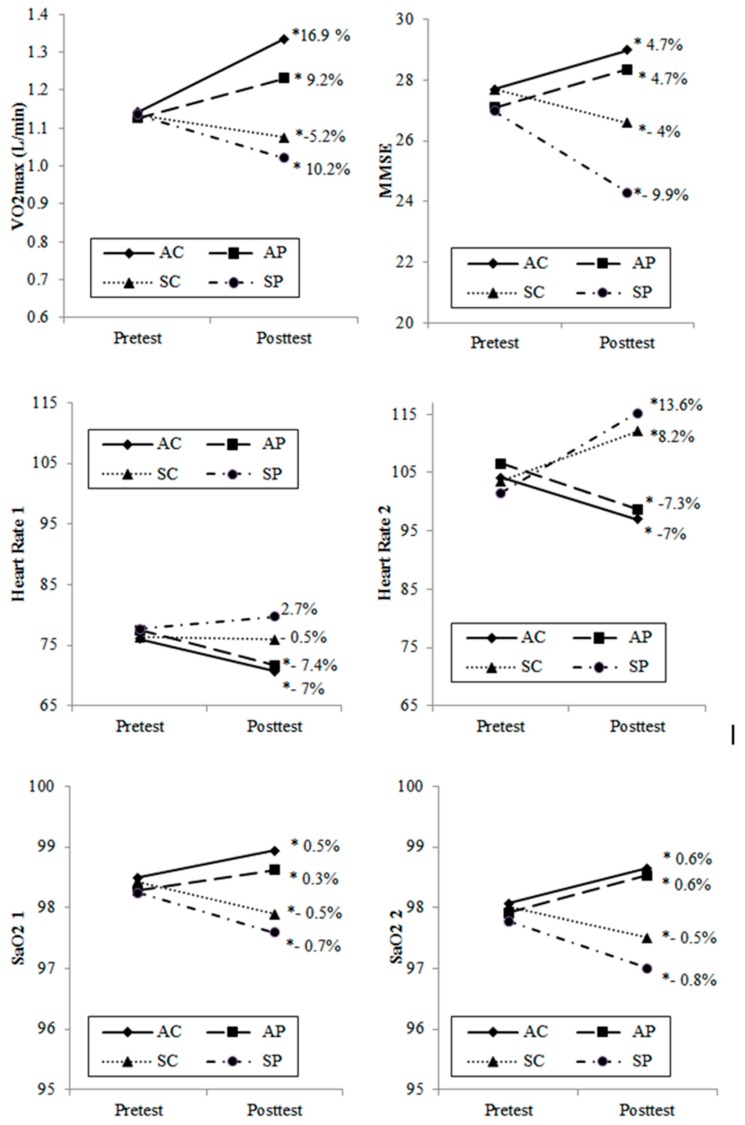
Intragroup absolute and relative change between post and pretest. AC: active/clean air group; AP: active/polluted air group; SC: sedentary/clean air group; SP: sedentary/polluted air group. * Significant differences (*p* < 0.05) between post and pretest (Wilcoxon or *t*-test according to normality).

Figure 2 shows the results obtained from the intragroup comparisons. These enable interpreting the evolution of each group throughout the two-year period of the experiment. The two groups that underwent a training program significantly improved all the variables analyzed, even the active group of elders who lived in an environment of polluted air improved both their physiological records and their cognitive states. On the other hand, the groups that followed a sedentary lifestyle throughout the two-year period of the experiment showed significantly worse results in all the variables analyzed, except for HR at rest, in which these changes were not important.

## 4. Discussion

The aim of this study was to analyze the effects of a monitored training program of cardiorespiratory exercise on cognition in older women living in areas of high air pollution compared with older women living in areas without air pollution. Table 2 shows the triennial average of air quality in the study areas, and the reference values recommended by both the World Health Organization (WHO) [25] and the Chilean authorities. It is noticeable that the Chilean regulations on air quality are permissive regarding the presence of atmospheric pollutants such as gaseous chemical compounds (NO_2_, O_3_, and SO_2_) and breathable particulate matter of diameter ≤2.5 and ≤10 µm (PM PM_2.5_ and PM_10_, respectively) which are pollutant values that exceed the levels recommended by the WHO. Likewise, the air quality in the areas where the participants lived recorded levels of particulate matter above the Chilean regulations. It has been reported that high levels of PM_10_ in the urban environment, where people live and work, increase the risk of respiratory disease [26] and acute mortality for all causes [27]. However, there are few studies in the literature that have analyzed the influence of these pollutants on neurocognitive disorders [8] and, although some cognitive deficiencies and behavioral alterations in children and elder people have been reported, these results are not conclusive due to the limited number of studies, small sample size or methodological limitations [28].

Table 3 shows intergroup comparisons before the experiment (Pretest). There are no statistically significant differences between the groups (*p* > 0.05). Therefore, it can be stated that all four groups started at a similar situation in all the variables analyzed.

### 4.1. Cognitive State Variables

Table 4 shows the differences between the groups in the posttest. Regarding the cognitive performances assessed by the MMSE, the results indicate that the group with a sedentary lifestyle, permanently exposed to air pollution (SP), obtained the lowest scores in the MMSE. Figure 2 shows the intragroup pre-post changes. At the end of the experiment, a statistically significant decreasing rate (*p* < 0.05) was observed in the SC and SP groups (−4% and −9.9%, respectively). This was in addition to significant differences (*p* < 0.05) at the end of the experiment between these two groups (Table 4). These results suggest that the greater loss of cognitive functions in the SP group could be attributed to the noxious effects of air pollution on mental health, which could lead to a precocious diagnosis of mild cognitive impairment according to Lourenço and Veras [20]. It has also been reported that there is a relationship between automobile-generated air pollution and cognitive function impairment in older men [29]. Even an association between the concentrations of PM_2.5_ particles and cognitive functions in older people has been demonstrated, concluding that an improvement in air quality may be an important strategy to reduce age-related cognitive impairment [5].

Despite all of the above-mentioned about the harmful effects of air pollution on cognitive functions, in the two active groups (AC and AP) who performed a program of progressive walk significant increases (*p* < 0.05) were recorded for intragroup changes in MMSE scores, 4.7% in both groups (Figure 2). In this respect, no statistically significant differences were found between the two active groups in the MMSE (Table 4), which suggests that cognitive functions in older people can be improved with aerobic training, regardless of the pollution factor. The length of the experiment (24 months, three training sessions per week) and the adherence to exercise obtained in the present study may account for the differences in the results obtained. These findings highlight the importance of active lifestyles concerning the effects of permanent exposure to air pollution. On the other hand, previous evidence suggests that reduced aerobic fitness is associated with accelerated cognitive impairment, which emphasizes the possible importance of implementing programs of physical exercise to optimize brain functions in senescence [30].

Executive functions of cognition in their different dimensions were measured through the MMSE; intergroup comparisons are presented in Table 3 and Table 4. It was observed that most of the significant differences between the groups in the posttests can be attributed to the physical exercise factor. It has been reported in previous studies that the habit of physical exercise, such as walking, may help to improve cognitive functions in patients with subcortical vascular cognitive impairment [31]. This also confirms that regular physical activity, three days per week, would improve brain functions in older people with mild cognitive impairment [32]. The neurotoxic effects of air pollution seem to be more obvious in older women with sedentary habits. According to the results found in the present study, these mainly affect the executive functions of computing capacity and temporal Orientation.

### 4.2. Physiological Variables

The groups of sedentary women (SC and SP), scored a lower cardiorespiratory performance (VO_2max_) with respect to the active groups at the end of the experiment (Table 4). In the intragroup changes between the beginning and the end of the experiment there was a statistically significant decrease (*p* < 0.05) of VO_2max_ of −5.2% and −10.2% in the SC and SP groups, respectively (Figure 2). Previous studies indicate that the decrease of aerobic capacity in older people may be due, among other factors, to a decrease and poor distribution of cardiac output, to a reduced oxidative capacity of skeletal muscle [33], to a limited mitochondrial content/mitochondrial dysfunction due to age [34], or to a decrease of the pulmonary function indices due to air pollution [35].

In the between group comparisons at the end of the experiment there were significant differences (*p* < 0.05) in VO_2max_ between the groups of active women with or without air pollution (AC/AP), indicating that the presence of air pollution caused a lower cardiorespiratory performance (Table 4). The women of the AC group showed the greatest increase, 16.9%, in VO_2max_ between the pretest and posttests, and the AP group obtained a lower increase, of 9.2%. In both groups the increases recorded were statistically significant (*p* < 0.05) (Figure2). Previous studies have reported improvements in aerobic capacity as a response to a monitored program of physical exercise in older people [36], in addition to the fact that maintaining regular physical activity produces a higher level of aerobic fitness [37]. 

On the other hand, the sedentary group (SP) showed significantly lower oxygen consumption values (*p* < 0.05) than the active group (AP), which demonstrates a higher cardiorespiratory tolerance to submaximal effort in the latter (AP). These results are not consistent with previous studies which reported that permanent exposure to environmental pollution increases cardiovascular risk factors [38] and is associated with accelerated biological aging [39], which significantly reduces the performance at submaximal levels of physical exertion [40]. However, these finds are in line with a study that supports the relationship between cardiorespiratory fitness and brain activity in patients with and without Alzheimer´s disease [16]. Likewise, it has been proposed that a good cardiorespiratory response is a good indicator of cognitive improvements, with aerobic exercise being a potentially important target therapy to achieve cognitive benefits [11]. 

On the other hand, it has been reported that an increase of oxygen saturation in blood leads to a decrease of the heart rate and a better memory performance [41]. It has also been observed that a good supply of oxygen may increase SaO2 in older people, enhancing their cognitive performance [42]. For both inactive groups, Figure 2 shows a decrease of peripheral oxygen saturation at rest after the two-year experiment (−0.5% and −0.7% for SC and SP, intragroup comparison, respectively). The differences between these two groups at the end of the experiment were not statistically significant in pairwise comparison (Table 4), which suggests that the effects of sedentary lifestyles, as a common factor, determine arterial oxygenation, regardless of being exposed to clean or polluted air. On the other hand, the two active groups show a statistically significant increase (0.6%; *p* < 0.05) of peripheral oxygen saturation, measured immediately after the effort, between the beginning and the end of the experiment (Figure 2). These results suggest that an active lifestyle favors the peripheral oxygen saturation and hence cognitive performance. These results are consistent with previous studies [43], [44].

It has also been observed that inhalation of particulate matter (PM) is associated with a rapid increase of blood pressure and heart rate (HR), probably due to imbalances in the autonomic nervous system (ANS), which would contribute to the activation of acute cardiovascular events [45]. Furthermore, it has been demonstrated that the presence of particulate matter (PM_10_, PM_2.5_) and nitrogen dioxide inside houses and in the outside environment is linked with an increase of HR [46]. However, on the other hand, it has also been mentioned that exposure to concentrations of PM in the air would cause a slight decrease of HR [47]. Figure 2 shows a significant decrease of HR-1 at rest in the two active groups, at the end of the experiment (AC: −7.4%, *p* < 0.05 and AP: −7.0%, *p* < 0.05; pre-post intragroup comparison). In intergroup comparisons of HR at rest at the end of the experiments (HRpost1) no significant differences were observed between the active groups, although there were considerable differences between the active groups and the sedentary groups (Table 4). It could be inferred that active life styles based on regular, systematic and planned physical exercise, regardless of the presence of breathable PM in the air, would cause a decrease of HR in older people who live in areas of high environmental pollution, despite the fact that increases of PM_10_ have been related to increases of HR. The two active groups showed similar decreases in HR (−7.0% and −7.3% in AC and AP, respectively) after submaximal exercise when comparing pretests (HRPre-2) with posttests (HRPost-2) (Figure2). Nevertheless, HR after submaximal effort between the two active groups at the end of the experiment was not significantly different (Table 4), which demonstrates that physical exercise was a more determining factor than air pollution. Finally, at the end of the experiment, the sedentary group that lived in areas of high air pollution (SP) showed the highest HR, both at rest (HRPost1) and after submaximal effort (HRPost2), compared to the other three groups (Table 4). This supports the idea that sedentary habits, along with air pollution, together with the typical involuntary efforts of daily life, which could be of a similar intensity to those performed in the present study, could induce cardiac fatigue. It has been noticed that an abnormal heart rhythm could be one of the mechanisms to explain the increase of strokes and cardiac events observed during the days of high air pollution [48], especially in older people with coronary artery disease [49].

## 5. Conclusions and Perspectives

The results obtained about the effects of cardiorespiratory training on cognitive functions and cardiovascular markers in older women demonstrate that, in order to maintain such functions at an optimal level, one must adopt an active lifestyle and live in a clean air environment. Likewise, active life habits based on regular exercise on days of better air quality help to decrease the toxic effects of air pollution in those older women who live in areas of high environmental pollution. On the other hand, a sedentary lifestyle and air pollution have a noxious effect on cognitive impairment in older women. These findings suggest that planned, regular, and systematic cardiorespiratory activity has a positive influence on brain integrity and on the mechanisms of oxygen transport, irrespective of the levels of air pollution. Therefore, active lifestyles could be a protective mechanism for brain and cardiovascular function in older women who are permanently exposed to atmospheric pollution. However, further research is needed to expand the knowledge and understanding of the protective effects that planned physical activity has on cognitive function and cardiovascular adaptations in the presence of high concentrations of particulate matter in the atmosphere.

## Figures and Tables

**Table 1:** Exercise protocol (horizontal walking). Session type (three per week) for each training cycle (4 months). VO_2max_: maximum oxygen uptake

Cycle	Training Protocol									Training Intensity			
	Exercise Time (minute) (VO_2max_ (%))										Metabolic Equivalents (METs)	Speed (km/h)	Caloric Expenditure (kcal/minute)
Cycle 1	10 (40)	5 (50)	10 (40)	5 (50)	Rest 5 minute	5 (40)	10 (50)	5 (40)	10 (50)		1.9–2.4	2–3	2.3–2.9
Cycle 2	10 (40)	5 (50)	10 (40)	5 (50)	Rest 5 minute	5 (40)	10 (50)	5 (40)	10 (50)		2.4–2.9	3–4	2.9–3.5
Cycle 3	10 (60)	5 (70)	10 (60)	5 (70)	Rest 5 minute	5 (60)	10 (70)	5 (60)	10 (70)		3.4–3.6	4–5	3.5–4
Cycle 4	10 (70)	5 (75)	10 (70)	5 (75)	Rest 5 minute	5 (70)	10 (75)	5 (70)	10 (75)		3.4–3.6	5–5.5	4–4.3

Training session duration 60 minutes.

**Table 2:** Registration of three-yearly average concentrations (2012–2013–2014) and reference limit values of the World Health Organization (WHO) and Chile.

Reference	PM_10_ µg/m^3^	PM_2.5_ µg/m^3^	NO_2_ µg/m^3^	O_3_ µg/m^3^	SO_2_ µg/m^3^
Pudahuel	63.8	28.0	38.9	* 112	NI
Viña del Mar	34.3	12.8	NI	NI	NI
Chile	** 50	** 20	** 100	* 120	80/24 h
WHO	** 20	** 10	** 40	* 100	20/24 h

PM_10_: particulate material diameter ≤10 µm; PM_2.5_: particulate material diameter ≤2.5 µm; NO_2_: nitrogen dioxide; O_3_: ozone; SO_2_: sulfur dioxide; NI: no information. * Percentile (P99) of the daily maximum 8 hours. **Annual average. Source: Sistema de Información Nacional de Calidad del Aire-Chile (SINCA)

**Table 3:** Intergroup comparisons pretest *.

	Active Clean (*n* = 45)			Active Pollution (*n* = 47)			Sedentary Clean (*n* = 45)			Sedentary Pollution (*n* = 44)			Intergroup Comparisions ^§^		
	M ± SD	CI (95%)		M ± SD	CI (95%)		M ± SD	CI (95%)		M ± SD	CI (95%)		F	*p-*value	Partial Eta ^2^
VO_2max_^(a)^	1.14 ± 0.1	1.12–1.16		1.13 ± 0.1	1.11–1.15		1.13 ± 0.06	1.12–1.15		1.14 ± 0.07	1.12–1.16		0.447	0.720	0.01
HRPre-1 ^(a)^	76.1 ± 1.9	74.5–77.7		77.5 ± 4.9	76–79.1		76.3 ± 3.4	74.7–77.9		77.7 ± 8.9	76.1–79.3		1.081	0.358	0.02
HRPre-2 ^(a)^	104.2 ± 8.2	101.3–107.1		106.5 ± 8.9	103.7–109.4		103.6 ± 10.7	100.7–106.4		101.5 ± 11.1	98.6–104.4		2.081	0.104	0.03
SaO_2_Pre-1 ^(a)^	98.5 ± 0.5	98.3–98.7		98.3 ± 0.6	98.1–98.4		98.4 ± 0.5	98.2–98.6		98.3 ± 0.8	98.1–98.4		1.595	0.192	0.03
SaO_2_Pre-2 ^(a)^	98.1 ± 0.5	97.8–98.3		97.9 ± 0.8	97.7–98.1		98.1 ± 0.7	97.8–98.2		97.8 ± 0.8	97.6–97.9		1.545	0.204	0.03
Time Orientation ^(b)^	4.87 ± 0.34	4.78–4.96		4.85 ± 0.36	4.76–4.94		5.0 ± 0.0	4.91–5.09		4.86 ± 0.35	4.77–4.95		2.41	0.07	0.04
Spatial Orientation ^(b)^	4.91 ± 0.36	4.81–5.02		4.91 ± 0.35	4.81–5.02		4.98 ± 0.15	4.87–5.08		4.82 ± 0.50	4.71–4.93		1.49	0.22	0.02
Registration ^(b)^	2.96 ± 0.21	2.89–3.02		2.98 ± 0.15	2.92–3.04		2.98 ± 0.15	2.92–3.04		2.95 ± 0.30	2.89–3.02		0.19	0.91	0.00
Calculation ^(b)^	4.04 ± 1.26	3.66–4.43		3.74 ± 1.48	3.37–4.12		3.89 ± 1.15	3.50–4.28		3.77 ± 1.34	3.38–4.16		0.49	0.69	0.01
Recall ^(b)^	2.76 ± 0.48	2.60–2.91		2.66 ± 0.56	2.51–2.81		2.73 ± 0.45	2.58–2.89		2.61 ± 0.58	2.46–2.77		0.71	0.55	0.01
Language ^(b)^	8.16 ± 0.85	7.88–8.43		8.00 ± 0.93	7.73–8.27		8.13 ± 1.01	7.86–8.41		7.95 ± 0.91	7.68–8.23		0.51	0.68	0.01
MMSE ^(b)^	27.7 ± 1.82	27.1–28.3		27.1 ± 2.19	26.5–27.8		27.7 ± 2.0	27.1–28.3		26.98 ± 2.4	26.3–27.6		1.41	0.24	0.02

^(a)^ Physiological variables and ^(b)^ cognitive state variables. * Mean ± standard deviation (M ± SD) and 95% confidence interval (CI). Effect size by partial Eta ^2^. ^§^ GLM multivariate analysis. One factor (group). Post hoc pairwise comparisons with Bonferroni adjustment (no significant differences between groups). MMSE: Mini Mental State Examination.

**Table 4:** Intergroup comparisons posttest *.

	Active Clean (*n* = 45)			Active Pollution (*n* = 47)			Sedentary Clean (*n* = 45)			Sedentary Pollution (*n* = 44)			Intergroup Comparisions ^§^		
	M ± SD	CI (95%)		M ± SD	CI (95%)		M ± SD	CI (95%)		M ± SD	CI (95%)		F	*p*-value	Partial Eta ^2^
VO_2max_^(a)^	1.34 ± 0.0 ^abc^	(1.33–1.35)		1.23 ± 0.1 ^ade^	(1.21–1.25)		1.08 ± 0.1 ^bdf^	(1.05–1.10)		1.02 ± 0.1 ^cef^	(0.99–1.05)		160.1	0.000	0.73
HRPost-1 ^(a)^	70.8 ± 5.8 ^ab^	(69.05–72.51)		71.8 ± 7.8 ^cd^	(69.5–74.1)		75.9 ± 5.1 ^ace^	(74.4–77.4)		79.8 ± 7.1 ^bde^	(77.6–81.9)		17.6	0.000	0.23
HRPost-2 ^(a)^	97.0 ± 3.1 ^ab^	(96.02–97.89)		98.7 ± 5.3 ^cd^	(97.2–100.3)		112.0 ± 9.4 ^ac^	(109.2–114.9)		115.3 ± 8.4 ^bd^	(112.8–117.9)		79.4	0.000	0.57
SaO_2_Post-1 ^(a)^	98.9 ± 0.3 ^abc^	(98.86–99.01)		98.6 ± 0.6 ^ade^	(98.4–98.8)		97.9 ± 1 ^bd^	(97.6–98.2)		97.6 ± 0.9 ^ce^	(97.3–97.9)		31.8	0.000	0.35
SaO_2_Post-2 ^(a)^	98.6 ± 0.5 ^ab^	(98.50–98.79)		98.5 ± 0.5 ^cd^	(98.4–98.7)		97.5 ± 1.3 ^ac^	(97.1–97.9)		97.0 ± 1.3 ^bd^	(96.6–97.4)		29.6	0.000	0.33
Time Orientation ^(b)^	4.96 ± 0.21 ^a^	(4.76–5.15)		4.83 ± 0.38 ^b^	(4.64–5.02)		4.80 ± 0.50 ^c^	(4.61–4.99)		4.18 ± 0.71 ^abc^	(3.99–4.37)		12.70	0.000	0.177
Spatial Orientation ^(b)^	5 ± 0.0 ^a^	(4.87–5.13)		5 ± 0.0 ^b^	(4.88–5.12)		4.84 ± 0.42	(4.72–4.97)		4.73 ± 0.44 ^ab^	(4.60–4.86)		4.27	0.006	0.068
Registration ^(b)^	2.97 ± 0.15 ^a^	(2.87–3.08)		2.98 ± 0.14 ^b^	(2.88–3.08)		2.76 ± 0.57 ^ab^	(2.65–2.86)		2.86 ± 0.36	(2.76–2.97)		4.28	0.006	0.068
Calculation ^(b)^	4.58 ± 0.81 ^ab^	(4.23–4.93)		4.17 ± 1.03 ^c^	(3.83–4.51)		3.84 ± 1.11 ^ad^	(3.50–4.19)		2.68 ± 1.37 ^bcd^	(2.33–3.03)		20.99	0.000	0.262
Recall ^(b)^	2.96 ± 0.21 ^ab^	(2.80–3.11)		2.89 ± 0.31 ^cd^	(2.74–3.04)		2.56 ± 0.69 ^ac^	(2.40–2.71)		2.59 ± 0.55 ^bd^	(2.44–2.75)		6.97	0.000	0.106
Language ^(b)^	8.53 ± 0.79 ^ab^	(8.22–8.84)		8.49 ± 0.59 ^cd^	(8.19–8.79)		7.80 ± 1.20 ^ac^	(7.49–8.11)		7.27 ± 1.16 ^bd^	(6.96–7.58)		14.81	0.000	0.201
MMSE ^(b)^	29 ± 1.3 ^ab^	(28.3–29.6)		28.4 ± 1.5 ^cd^	(27.7–29)		26.6 ± 2.1 ^ace^	(25.9–27.2)		24.32 ± 2.85 ^bde^	(23.7–24.9)		39.80	0.000	0.403

^(a)^ Physiological variables and ^(b)^ cognitive state variables. * means ± standard deviation (M ± SD) and 95% confidence interval (CI). Effect size by partial Eta ^2^. ^§^ GLM multivariate analysis. One factor (group). ^a,b,c,d,e,f^. Post hoc pairwise comparisons, Bonferroni adjustment. The same superscripts (^a^, ^b^, ^c^, ^d^, ^e^, ^f^) in values indicate significant differences between groups.
